# A comparative evaluation of EEG Power spectrum characteristics between two methods for alleviating preoperative anxiety in breast cancer patients

**DOI:** 10.3389/fpsyt.2025.1611129

**Published:** 2025-07-25

**Authors:** Xing Jin, Yuxing Wang, Hui Jiang, Ying Wu, Yi Liu

**Affiliations:** ^1^ Department of Anesthesiology, Shanxi Province Cancer Hospital/Shanxi Hospital Affiliated to Cancer Hospital, Chinese Academy of Medical Sciences/Cancer Hospital Affiliated to Shanxi Medical University, Taiyuan, China; ^2^ School of Humanities and Social Sciences, Research Center for Psychological and Health Sciences, Shanxi Medical University, Taiyuan, China

**Keywords:** EEG power spectrum, preoperative anxiety, psychological intervention, midazolam, network analysis

## Abstract

**Introduction:**

Women with breast cancer are prone to moderate to severe preoperative anxiety. Effective measures for preventing and managing preoperative anxiety include drug therapy and non-drug intervention. The main evaluation method is scale assessment, which has certain limitations and may involve human concealment or evaluation errors. In this study, resting-state electroencephalography (EEG) was used to explore changes in power spectrum during the alleviation of preoperative anxiety in breast cancer patients, which is of great significance for objectively identifying and evaluating preoperative anxiety in patients.

**Methods:**

40 breast cancer patients were randomly divided into two groups (20 patients per group), receiving either psychological intervention (PI) or intravenous midazolam (MID) before surgery. Visual Analog Scale for Anxiety (VAS-A) and Observer’s assessment alert/Sedation (OAA/S) scores, clinical monitoring indexes and EEG data were measured before and after intervention.

**Results:**

VAS-A scores significantly decreased in both groups (p < 0.05), the power in the theta band of the frontal parietal regions decreased (p < 0.05) and was positively correlated with VAS-A scores (p < 0.001). Network analysis revealed that the three highest centrality measures in the PI group were located in the alpha band frontal parietal region, frontal central region, and gamma band parietal region, while the OAA/S scores showed the highest centrality for all three measures in the MID group.

**Conclusions:**

Both PI and intravenous MID can effectively alleviate preoperative anxiety in breast cancer patients, but their neuroelectrophysiological mechanisms were not entirely the same. Regarding the relationship between brain region power and monitoring indexes, the power of three specific regions in certain frequency bands was the primary factor in the PI group, while the level of sedation was the determining factor in the MID group.

## 1 Introduction

The 2020 Global Cancer Report indicated that breast cancer, with 2.3 million new cases, has surpassed lung cancer to become the leading cause of cancer worldwide (1). It is also the most prevalent cancer among women, accounting for up to 30% of all cancer cases in this group, and is the primary cause of death among female cancer patients (2). Surgery is one of the primary treatments for breast cancer, but this therapeutic approach can trigger a series of psychological reactions, with preoperative anxiety being the most typical response. Relevant data shows that 11% to 80% of adult surgical patients experience preoperative anxiety to varying degrees (3). Those experiencing anxiety may feel tense, worried, fearful, and anxious. Appropriate anxiety is a normal reaction to adapt to environmental changes, but excessive anxiety can interfere with the smooth progression of anesthesia and surgery, often leading to increased heart rate, elevated blood pressure, and even arrhythmias, which can disrupt the initiation or completion of surgery, and in severe cases, may result in surgery being suspended or canceled (4). Excessive anxiety can also lead to excessive consumption of body energy and tissue breakdown, weakened overall regulatory function of the body, and decreased immune function, thereby increasing the risk of complications and affecting the effectiveness of surgical treatment. High levels of preoperative anxiety not only cause discomfort for patients before surgery but can also result in postoperative psychosocial changes, manifesting as a decreased pain threshold, increased need for analgesics, prolonged recovery and hospitalization times, and decreased satisfaction and trust in healthcare providers. Therefore, preoperative anxiety has a significant impact on various aspects of surgical procedures. Adopting effective management strategies to alleviate preoperative anxiety can accelerate postoperative recovery, shorten hospitalization time and costs, enhance patient trust, improve doctor-patient relationships, and increase patient satisfaction with hospitalization.

Female patients are at a higher risk of experiencing moderate to severe preoperative anxiety (5). When confronted with significant events such as surgery, female patients are prone to anxiety, fear, and other negative emotions, and are more likely to develop moderate to severe anxiety (6). Currently, effective intervention measures for the prevention and management of preoperative anxiety include pharmacological treatments and non-pharmacological interventions. Benzodiazepines, for instance, are a common and effective pharmacological intervention for reducing preoperative anxiety (7). Research results indicate that preoperative administration of 0.06 mg/kg midazolam (MID) can significantly reduce patients’ mental stress, although physical fatigue may be more pronounced, it remains within the normal range, which may be related to the sedative effects of MID (8). However, anti-anxiety medications can produce adverse effects such as respiratory depression, nausea and vomiting, gastrointestinal motility disorders, drowsiness, delirium, and allergies (9). Therefore, clinical healthcare professionals have begun to explore non-pharmacological methods to alleviate patients’ anxiety and have achieved good results (10, 11). Psychological intervention (PI) also plays a crucial role in the prevention of preoperative anxiety. Studies have shown that providing patients with 30 minutes of emotional support therapy preoperatively can improve their preoperative anxiety (12), supportive psychotherapy has significantly reduced anxiety, depression, and other negative emotions in patients with gastrointestinal malignancies, enhancing their postoperative independent living abilities and quality of life (13). A randomized controlled study revealed that, compared to the conventional control group, the experimental group that received mindfulness-based stress reduction therapy showed lower scores for anxiety and depression, as well as shorter hospital stays and lower hospitalization costs (14).

The primary method for assessing preoperative anxiety among breast cancer patients is through the use of rating scales, such as the Beck Anxiety Inventory (BAI), the Hamilton Anxiety Scale (HAMA), and the Self-Rating Anxiety Scale (SAS), which are widely employed. However, these scales have different limiting conditions and scopes of application (15, 16). They tend to assess anxiety from a subjective perspective and, while convenient to use, have certain limitations. Individuals may control their external expressions and conceal their true thoughts through increased or decreased verbal and physical actions, habitual rational thinking, etc., which may lead to intentional concealment or assessment errors.

With the development of biomedicine, more and more studies have discovered objective physiological indicators of anxiety and depressive moods. The clinical application of brain functional imaging techniques is becoming increasingly widespread. By utilizing these techniques, doctors can objectively and clearly obtain changes in physiological signals related to the patient’s brain, greatly enhancing the reliability and accuracy of medical diagnoses. Techniques such as electroencephalography (EEG) and functional near-infrared spectroscopy (fNIRS), which record and provide information on brain activation states, can assist doctors in the clinical diagnosis of brain dysfunction disorders and are widely used in research on depression identification, epilepsy detection, schizophrenia identification (17, 18), motor imagery, and more. Therefore, using objective indicators such as brain imaging to explore abnormal changes in brain function is of great significance for truly and objectively identifying and assessing preoperative anxiety in patients.

## 2 Materials and methods

### 2.1 Participants

This study selected female patients scheduled for elective breast cancer surgery under general anesthesia from January to March 2024. The inclusion criteria were: age 20–65 years; Body Mass Index (BMI) 18–28 kg/m²; American Society of Anesthesiologists (ASA) grade I-II; clear consciousness, normal cognition function, and ability to independently complete anxiety-related rating scales. Exclusion criteria included: history of neurological or psychiatric disorders; recent use (within 3 months) of sedative-hypnotic medications; significant impairment of major organ systems (including cardiac, hepatic, or renal dysfunction); hypersensitivity or allergy to MID. Participants were randomly allocated to either the PI group (n=20) or the MID group (n=20) in this randomized controlled trial. The sample size calculation, performed using PASS software, was based on preliminary data demonstrating that intravenous MID administration resulted in a reduction of Visual Analogue Scale for Anxiety (VAS-A) scores from 6.7 ± 2.0 to 5.4 ± 1.5. With the α level set at 0.05 (two-tailed) and power at 80%, the estimated required sample size was 19 patients per group. Considering 1:1 randomization, the study planned to include at least 38 participants (19 in each group). Ultimately, 40 patients were enrolled and randomly assigned to either the PI or MID group (20 patients each) using a random number table method. The allocation sequence was concealed in sequentially numbered, opaque sealed envelopes until intervention implementation, and outcome assessors remained blinded to group assignment throughout the study period.

The studies involving humans were approved by the Ethics Committee of Shanxi Medical University (2024021), and was conducted in accordance with the local legislation and institutional requirements. All participants were thoroughly briefed on the study aims, procedures, and their rights, and subsequently provided written voluntary informed consent.

### 2.2 Clinical measurement

All patients in the surgical waiting area of the operating room were assessed for anxiety using the Visual Analog Scale for Anxiety (VAS-A). The VAS-A consists of a 100-millimeter horizontal line, with one end labeled “Not anxious at all” (score 0) and the other end labeled “Extremely anxious” (score 10). The depth of sedation was determined by the Observer’s Assessment of Alertness/Sedation (OAA/S) scale. The OAA/S is a five-point scale used to assess responses to verbal stimulation, speech, facial expressions, and eye responses. The OAA/S measures levels of sedation ranging from 1 (no response to mild stimulation or shaking) to 5 (easily responds to their name spoken in a normal tone of voice). Additionally, patients’ vital signs, including heart rate (HR), mean arterial pressure (MAP), and percutaneous arterial oxygen saturation (SpO_2_), were continuously monitored.

The EEG data were obtained using a 32-channel device from Neuracle with a sampling rate of 1000 Hz for each patient, recording 10 minutes of resting-state EEG with eyes closed 15 minutes before and after the intervention (marked as time points T0 and T1). The channels on the scalp according to the international 10–20 system: Fp1, Fp2, Fz, F3, F4, F7, F8, FC1, FC2, FC5, FC6, Cz, C3, C4, T7, T8, CP1, CP2, CP5, CP6, Pz, P3, P4, P7, P8, PO3, PO4, Oz, O1, O2, with A1 and A2 as the reference electrodes.

### 2.3 Intervention procedures

PI Group. The PI protocol was meticulously developed by licensed clinical psychologists based on established cognitive therapy (CT) principles and progressive relaxation techniques (19), then standardized for implementation by trained anesthesiologists who underwent comprehensive training in the protocol. During the implementation process, the patient’s compliance was evaluated through interaction with the patient and the integrity of the process. Firstly, explain the disease knowledge, the necessity of surgery and anesthesia to the patients, so that they can fully understand and recognize the disease and surgical treatment. Clarify the significance of cooperating with the treatment and address their concerns in a targeted manner. Then, adopt a stable and slow method of deep inhalation and exhalation to achieve relaxation. Patients are generally required to breathe continuously for more than 10 minutes, with a respiratory rate of approximately 10–15 breaths per minute. When inhaling, patients should slowly clench their fists, slightly bend their wrists, hold their breath for a short period after taking a maximum inhale, and then exhale slowly, relaxing their hands and allowing their entire body’s muscles to be in a state of relaxation. Let their consciousness gently focus on their breath, experiencing the entire process of inhalation and exhalation. Afterwards, ask the patients to close their eyes and try to visualize some scenes that symbolize hope in their minds, such as the sun slowly rising in the morning or the starry night sky.

MID Group. Intravenous MID at a dose of 0.02 mg/kg was administered, and the patients were instructed to close their eyes and rest. If complications occur, including apnea (intermittent apnea > 10 seconds), arrhythmia, hypotension, or a drop in SpO_2_ value below 85%, they should be promptly treated. At the T0 and T1 time points, HR, MAP, SpO_2_, VAS-A score and OAA/S score were recorded, and the EEG data were collected.

### 2.4 EEG analysis

All EEG data were imported into the EEGLAB toolbox in MATLAB R2018b software. The bandpass filter band was 0.1–80 Hz, and the notch filter band range was 49–51 Hz. The continuous EEG was downsampled to 500 Hz, and the EEG data were segmented with 2-s epoch. Then, Spectral power analysis using Fast Fourier Transformation was applied on the scalp signals using the EEGLAB toolbox. Absolute powers were evaluated in six frequency bands: Delta (1–3 Hz), Theta (4–7 Hz), Alpha (8–12 Hz), Beta (13–30 Hz), Gamma (31–100 Hz). The scalp electrodes were clustered into nine groups according to the regions of interest (ROI), including the frontal parietal region (Fp1,Fp2), frontal region (Fz, F3, F4, F7, F8), frontal central region (FC1, FC2, FC5, FC6), central region(Cz, C3, C4), temporal region (T7, T8), central parietal region (CP1, CP2, CP5, CP6), parietal region(Pz, P3, P4, P7, P8), parietal occipital region (PO3, PO4), and occipital region (Oz, O1, O2). In this study, power spectral analysis was conducted using relative power for comparison (18), The relative power of each frequency band was obtained by dividing the absolute power of each band by the total bandwidth of absolute power.

### 2.5 Data analysis

The chi-square test was used for qualitative variables and the two-sample t-test or the Mann-Whitney test (depending on the normality of the distribution) for quantitative variables to compare demographic and clinical data. For the comparison of resting-state power in the ROIs between groups, an independent samples t-test was used, while for the within-group comparison before and after the intervention, a paired samples t-test was employed. Pearson’s correlation coefficient is used to assess the correlation between anxiety scores and the power of the ROIs in the brain for two groups of patients. Network analysis is used to evaluate the relationship between all monitored indexes and the power of the ROIs across various frequency bands. By applying the Least Absolute Shrinkage and Selection Operator (LASSO) for network regularization and setting the tuning parameter γ=0.5 to construct the network model, we can eliminate those messy and unstable connections, resulting in a stable and interpretable network. The importance of each node in the network is further investigated by examining the node’s strength, betweenness, and closeness.

## 3 Results

### 3.1 Differences in demographic characteristics and clinical monitoring indexes

The demographic characteristics of the patients are shown in **Table 1**. There were no statistical differences in age, education Duration, BMI and ASA grade between the two groups.

**Table 1:** The demographic characteristics of participants.

Characteristics	PI (n=20)	MID (n=20)	t/χ^2^	P
Age (years)	54.75(11.29)	52.45(10.85)	0.657	0.515
Education Duration (Years)	11.45(4.17)	11.65(4.75)	-0.141	0.888
BMI (kg/m^2^)	20.48(2.54)	23.76(2.08)	0.984	0.331
ASA(I/II)	3/17	4/16	0.173	0.677

Values are expressed as mean (SD) or number of patients.

The differences in clinical monitoring indexes are shown in **Table 2**. There were no statistically significant differences in hemodynamic parameters between the two groups at T0. In the PI group, MAP, OAA/S score, and VAS-A score at T1 were significantly lower than those at T0 (p < 0.05). In the MID group, MAP, SpO_2_, OAA/S score, and VAS-A score at T1 were significantly lower compared to T0 (p < 0.05). At the T1 time point, MAP, SpO_2_, and OAA/S score in the MID group were significantly lower than those in the PI group (p < 0.05).

**Table 2:** Comparison of monitoring indexes among participants.

Indexes	Group	T0	T1	t	P
MAP (mmHg)	PI	102.63 (8.18)	95.02 (7.23)	3.057	0.006
	MID	102.13 (7.91)	90.32 (6.44)^*^	5.673	0.000
HR (BMP)	PI	85.10 (12.16)	80.10 (11.19)	1.368	0.187
	MID	85.30 (13.24)	80.05 (11.30)	1.527	0.143
SpO_2_ (%)	PI	97.55 (1.00)	97.70 (0.92)	-0.513	0.614
	MID	97.55 (1.10)	95.85 (2.18)^*^	2.882	0.010
OAA/S	PI	5.00 (0.00)	4.60 (0.50)	3.559	0.002
	MID	5.00 (0.01)	3.75 (0.45)^*^	12.583	0.000
VAS	PI	5.75 (1.94)	3.15 (1.09)	5.319	0.000
	MID	5.60 (1.60)	3.25 (1.21)	5.702	0.000

Values are expressed as mean (SD); BMP, Beats Per Minute. * P < 0.05 compared with group PI.

### 3.2 Resting EEG power

**Figure 1:**
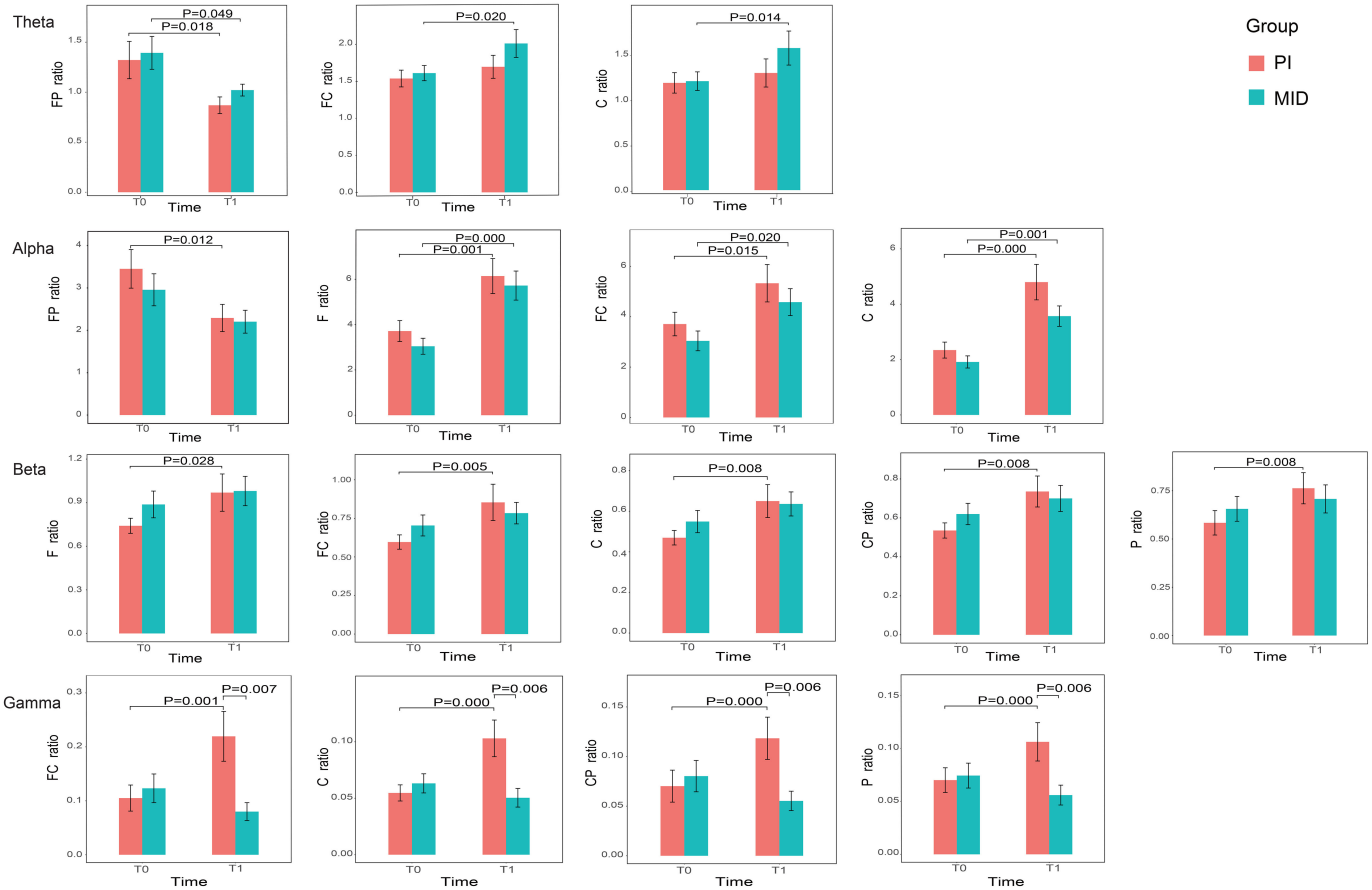
The relative power of the ROIs in each frequency band with statistically significant differences before and after intervention for two groups.

**Figure 1** shows the power and P-values of the ROIs in the brain for the two patient groups with statistically significant differences before and after the intervention during eye-closed conditions. For ease of description, the names of the ROIs are abbreviated, such as the frontal parietal region being abbreviated as FP, and the frontal parietal region in the Delta band being referred to as DeltaFP. In the Theta band: the power in FP for both the PI and MID groups significantly decreased at T1 compared to T0; the power in FC and C for the MID group significantly increased at T1 compared to T0. In the Alpha band: the power in FP for the PI group significantly decreased at T1 compared to T0; the power in the F, FC, and C for both the PI and MID groups significantly increased at T1 compared to T0. In the Beta band: the power in the F, FC, C, CP, and P for the PI group significantly increased at T1 compared to T0. In the Gamma band: the power in the FC, C, CP, and P for the PI group significantly increased at T1 compared to T0; at T1, the power in the FC, C, CP, and P for the MID group decreased significantly compared to the PI group.

### 3.3 Correlation analysis

**Figure 2:**
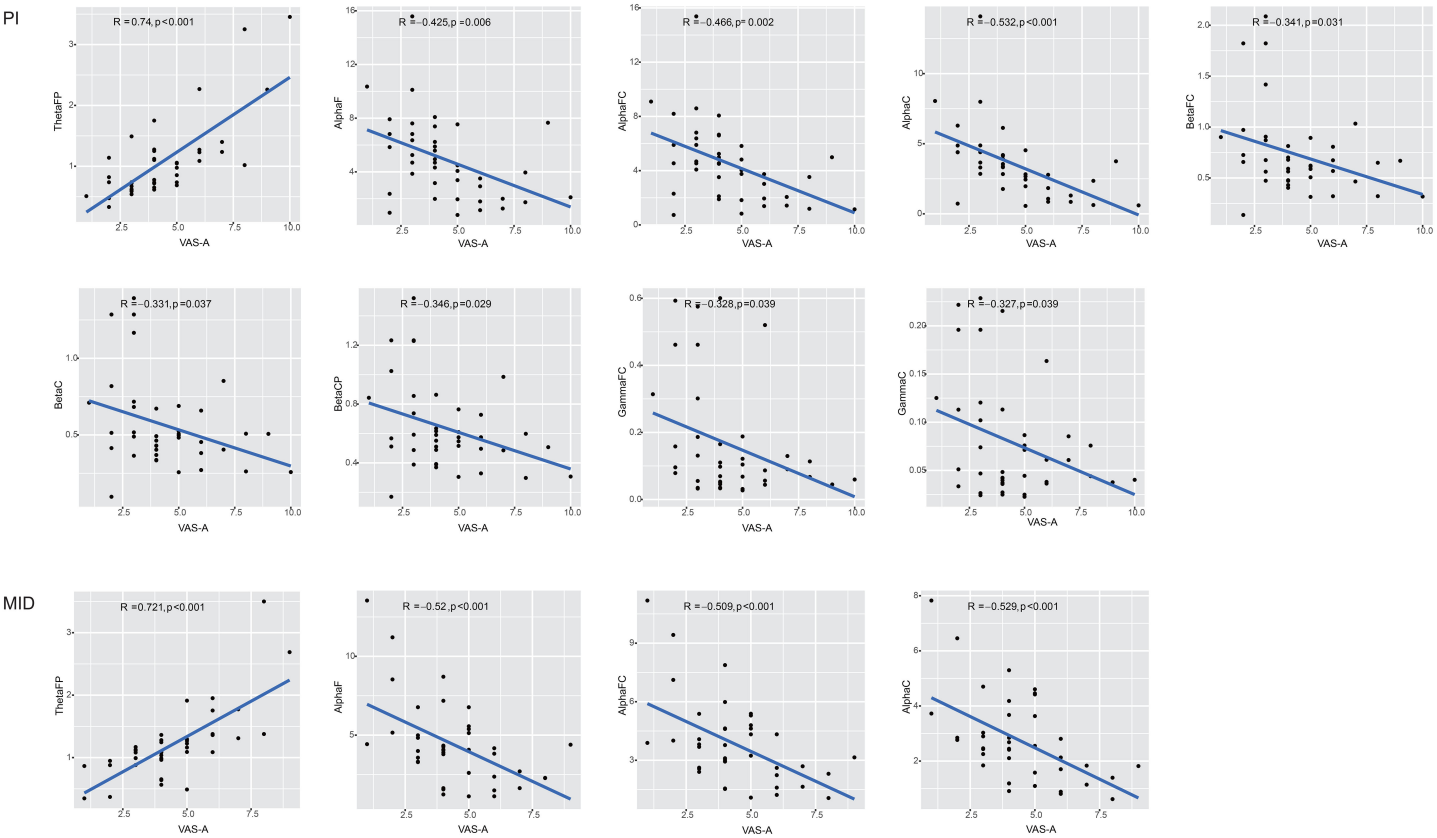
The correlation between the relative power of the ROIs and VAS-A scores.

A correlation analysis was conducted between the power of the ROIs in the brain, which showed statistically significant differences before and after the intervention within groups, and the VAS-A scores (**Figure 2**). In the PI group, the power of nine ROIs (ThetaFP, AlphaF, AlphaFC, AlphaC, BetaFC, BetaC, BetaCP, GammaFC, and GammaC) was correlated with the VAS-A scores, with the highest correlation coefficient observed for ThetaFP (R=0.74, p<0.001). In the MID group, the power of four ROIs (ThetaFP, AlphaF, AlphaFC, and AlphaC) was correlated with the VAS-A scores, with the highest correlation coefficient also observed for ThetaFP (R=0.721, p<0.001). The power of ThetaFP was positively correlated with the VAS-A scores in both groups, while the power of the other ROIs was negatively correlated with the VAS-A scores in both groups.

### 3.4 Network analysis

**Figure 3:**
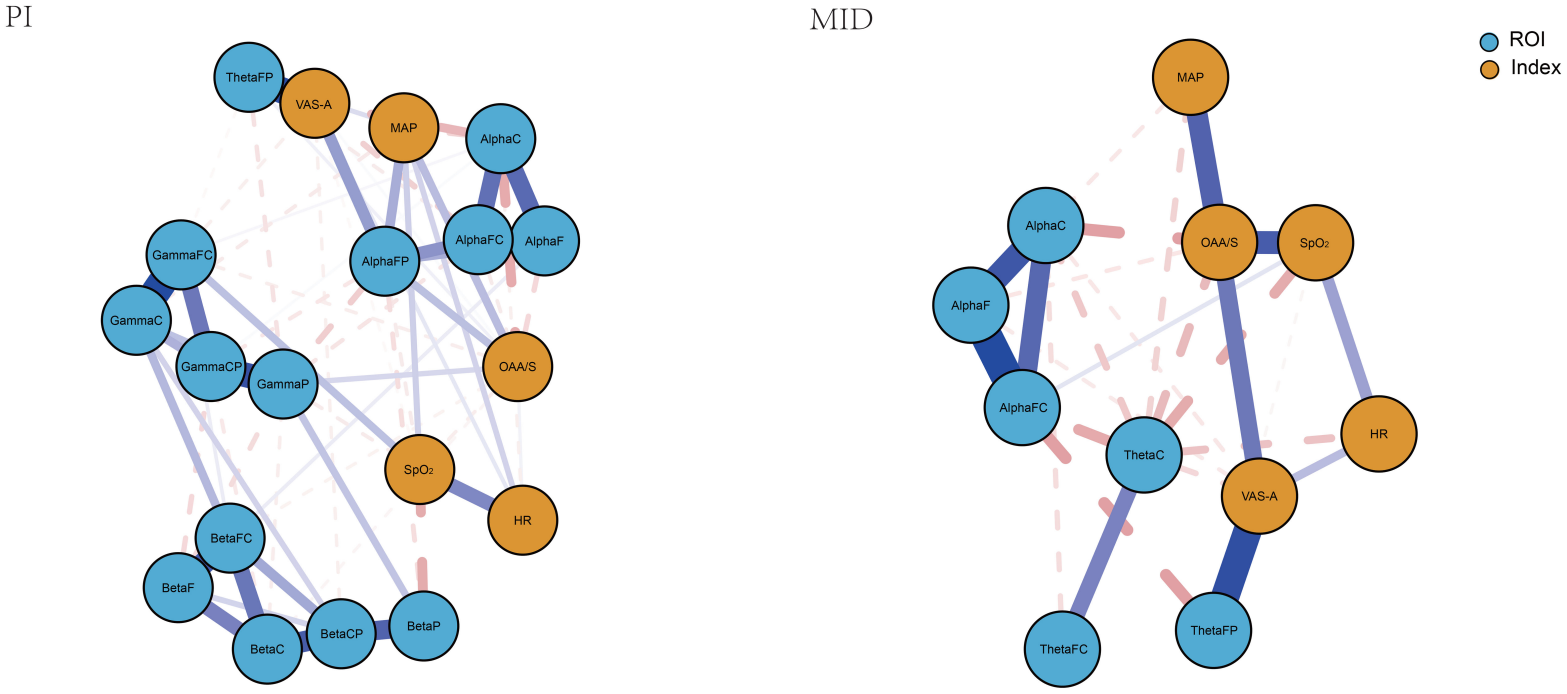
The network structure of the ROIs and monitoring indexes for two groups.

Using the estimateNetwork function from the bootnet package in R, a partial correlation coefficient network was constructed between the power of the ROIs in the brain that showed statistically significant differences before and after the intervention and all monitored indicators. Then, the LASSO method was used to shrink the edges in the network, making the network more concise and intuitive (20). As shown in **Figure 3**, each brain region or indicator was considered a node, with blue nodes representing ROIs and orange nodes representing monitored indexes. The associations between nodes were represented as edges. The closer the nodes were to each other and the thicker the edges, the stronger the correlation between the two nodes. Green edges indicated a positive correlation, while red edges indicated a negative correlation. In the PI group, from the distribution of ROIs, ROIs within the same frequency band were closer and more tightly connected, with positive correlations among them. ThetaFP and AlphaFP showed a closer and positive correlation with VAS-A compared to other brain regions. The distribution of monitored indexes was more dispersed, with a relatively weak positive correlation between VAS-A and MAP. In the MID group, ROIs within the same frequency band were closely connected with positive correlations. ThetaFP and ThetaC showed a closer relationship with VAS-A, with ThetaFP showing a positive correlation and ThetaC showing a weak negative correlation with VAS-A. The monitored indicators were centrally distributed around OAA/S, with VAS-A directly connected to OAA/S and HR, showing a positive correlation.

**Figure 4:**
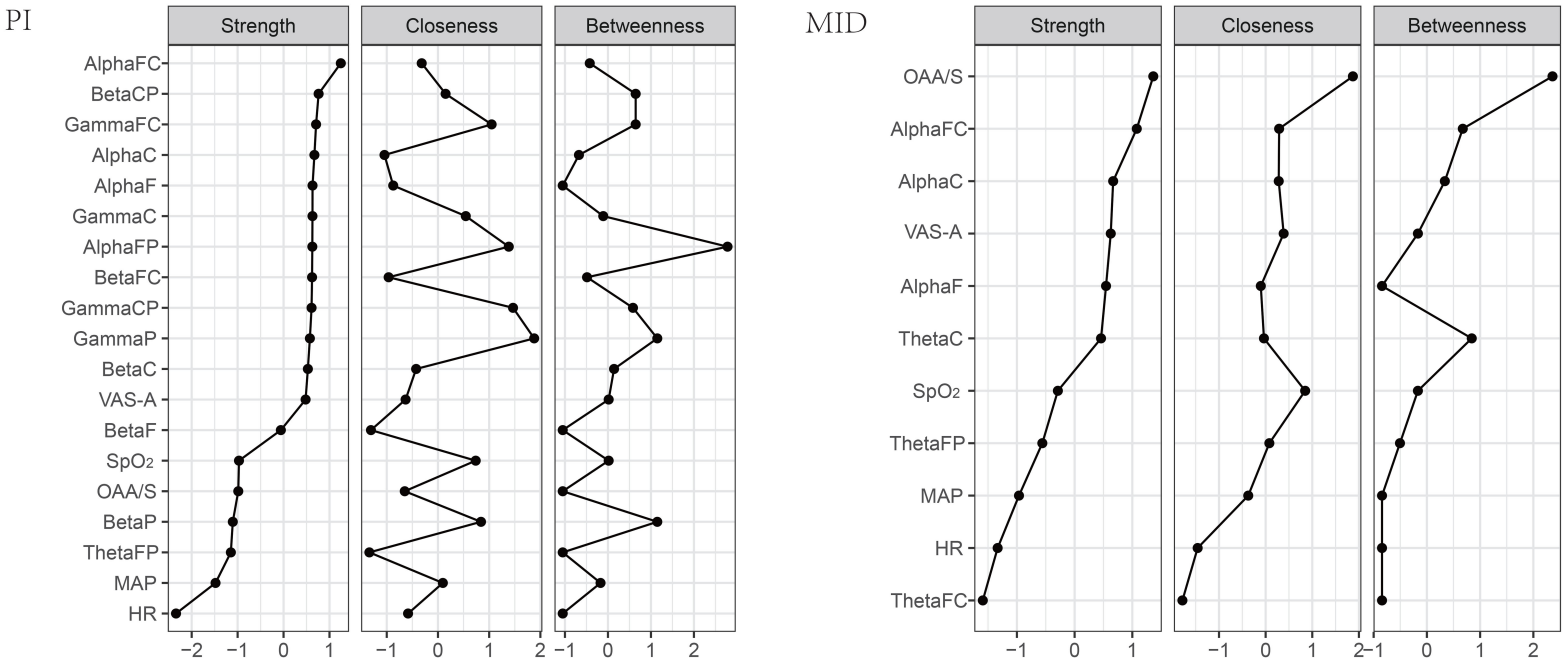
Comparison of network centrality indexes for two groups.

**Figure 4** compares the centrality indicators of the two networks. Strength refers to the total weight of connections between one node and all other nodes in the network, typically representing the most important measure that has the greatest impact on network stability. Closeness centrality measures the average shortest path length from a node to all other nodes, reflecting information transmission efficiency - nodes with the highest closeness can influence other nodes more rapidly. Betweenness centrality evaluates the importance of a node as a “bridge” by counting how many shortest paths pass through it, where nodes with high betweenness are those that facilitate connections within the network. For instance, a resting-state functional connectivity study revealed that Strength (Dnodal) and closeness centrality (Enodal) of the beta and gamma bands were decreased in patients with post-traumatic stress disorder (PTSD) compared to healthy controls. The decreased nodal centrality values were observed primarily at the frontocentral electrodes (21). In the PI group of this study, AlphaFC has the highest correlation strength, primarily connected to ROIs within the same frequency band, as well as to OAA/S and MAP among the monitored indexes, making it the most important node in the entire network. GammaP has the highest closeness centrality, located at the center of the entire network, associated with other ROIs and monitored indexes. AlphaFP has the highest betweenness centrality, with the most extensive connections to other nodes in the network. In the MID group, OAA/S has the highest values for all three centrality indicators.

## 4 Discussion

The results of clinical monitoring indexes show that both PI and intravenous injection of low-dose MID can effectively alleviate preoperative anxiety in breast cancer patients, which is consistent with previous research findings (22–24). Both methods also significantly reduce the patients’ level of sedation and MAP. However, there is a difference in that MID intervention led to a noticeable but clinically safe decrease in SpO2. PI can reduce the excitability of the sympathetic nervous system, thereby decreasing the secretion levels of hormones such as adrenaline and noradrenaline, and alleviating negative emotions such as anxiety and nervousness in patients, as well as reducing abnormal increases in blood pressure. On the other hand, MID’s effects of lowering arterial pressure, reducing SpO2, and increasing the level of sedation are determined by its pharmacological mechanism. It activates benzodiazepine receptors and interferes with the reuptake of gamma-aminobutyric acid (GABA), enhancing the binding of GABA to its receptors, thereby exerting anti-anxiety, sedative, and other effects (25).

The results of the power spectral analysis of EEG in the ROIs showed that in the PI group, there was no significant change in the power of the delta band across all brain regions after the intervention compared to before; in the theta band, the power of ThetaFP decreased, and there was an upward trend in the power of the central brain regions, although this was not statistically significant; in the alpha band, apart from a decrease in AlphaFP power, the power of the central brain regions increased significantly; in the beta and gamma bands, the power of the central regions as well as the frontal and parietal lobes increased significantly. A systematic review on the EEG of mindfulness meditation revealed that, compared to the eyes-closed resting state, mindfulness is most commonly associated with increased alpha and theta power, although these findings are not uniformly reported. No consistent patterns were observed in the beta, delta, and gamma bandwidths. The co-presence of increased alpha and theta power may indicate a relaxed yet vigilant state conducive to mental health (26). Some studies have also reported that mindfulness practice can increase gamma band power and elevate overall theta band power while reducing frontal lobe theta activity (27, 28). Therefore, the alleviation of anxiety in patients in this experiment may be related to the state of mindfulness. In the MID group, there was no significant change in delta band power across brain regions after the intervention compared to before; in the theta band, ThetaFP power decreased, while power in the central brain regions increased significantly; in the alpha band, power in the central brain regions and the frontal lobe increased significantly; there was no significant change in beta and gamma band power, but when compared to the PI group after the intervention, gamma band power in the central brain regions and the parietal lobe decreased significantly. Studies have suggested that the anxiolytic effect of MID is associated with an increase in low-frequency EEG spectral energy (29). The theta wave may dominate the EEG spectrum in patients under general anesthesia (30), and changes in theta wave spectral energy are related to the loss and recovery of consciousness during general anesthesia. Therefore, the changes in EEG power in the MID group may be related to its pharmacological effects. The decrease in ThetaFP power was a common feature of anxiety relief achieved by both methods.

The results of the correlation analysis between the EEG power spectrum in the ROIs and the VAS-A scores showed that in the PI group, the VAS-A scores were positively correlated with ThetaFP power and negatively correlated with the power in the frontal, frontal central, and central regions of the alpha band, as well as the frontal central and central regions of the beta and gamma bands. In the MID group, the VAS-A scores were also positively correlated with ThetaFP power and negatively correlated with the power in the frontal, frontal central, and central regions of the alpha band. It can be seen that the alleviation of anxiety in both patient groups was associated with a decrease in ThetaFP power and an increase in the power of the frontal, frontal central, and central regions of the alpha band.

Considering that in addition to the VAS-A scores, other monitoring indexes also showed statistically significant changes before and after the intervention in both patient groups, and corresponding changes were observed in the EEG power spectrum of the ROIs, this study employed network analysis to interpret the relationships between the EEG power in the ROIs and various monitoring indexes. Specifically, the network formed in the PI group was relatively complex, with the highest connection strength in AlphaFC, indicating that this region played a crucial role in maintaining the entire network. GammaP showed the highest closeness centrality, suggesting extensive and most convenient connections between this region and various brain regions and monitoring indexes. AlphaFP showed the highest betweenness centrality, indicating that various brain regions and monitoring indexes were primarily connected through this region. These three brain regions, as important nodes in the network, played a major role in the changes in the power of the ROIs and monitoring indexes in the PI group. Additionally, the relatively close relationship between VAS-A and MAP suggested that the decrease in MAP was mainly related to the alleviation of anxiety. The network in the MID group was relatively simple, with the highest values for all three centrality indicators for OAA/S, indicating that the changes in the EEG power of the regions of interest and monitored indexes in this group were mainly influenced by the level of sedation.

## 5 Limitation

The current study has several limitations that should be acknowledged. First, the sample size was relatively small, and the lack of long-term follow-up limits our ability to assess the persistence of observed EEG changes over time. Second, our analysis was restricted to resting-state EEG, without exploring functional connectivity or event-related potentials (ERPs), which might offer a more comprehensive understanding of neural alterations. Third, the network analysis relied on the LASSO method, which, despite its utility in feature selection, may introduce bias and influence the robustness of the findings. Additionally, the generalizability of our results is constrained by the exclusive focus on female breast cancer patients. Given the rarity of male breast cancer, our findings may not extend to this population. Future studies should include more diverse cohorts to validate the observed EEG patterns across different demographic and clinical groups. Moreover, further research is needed to determine whether these EEG biomarkers are applicable to patients undergoing other types of surgery, such as major abdominal or cardiac procedures, where postoperative mental state changes are also commonly reported.

## 6 Conclusions

In conclusion, both PI and intravenous injection of MID can effectively alleviate preoperative anxiety in breast cancer patients, but the neuroelectrophysiological mechanisms are not entirely the same. Firstly, the changes in the power spectrum of the ROIs in both patient groups may be related to the state of mindfulness or the pharmacological properties of MID. Secondly, the positive correlation between the power in the frontal parietal region of the theta band and anxiety scores is a common feature in both patient groups. Finally, in the complex relationship between the power of the ROIs and clinical monitoring indicators in the PI group, the power in the frontal-parietal region and frontal central region of the alpha band, and the parietal region of the gamma band play major roles. The sedative effect of MID is the primary factor determining the relationship between the power of the ROIs and monitoring indexes in the MID group.

## Data Availability

The raw data supporting the conclusions of this article will be made available by the authors, without undue reservation.
